# Taxonomic assessment of *Zhangixalus puerensis* (He, 1999) sensu lato (Anura, Rhacophoridae), with description of two cryptic species

**DOI:** 10.3897/BDJ.14.e204046

**Published:** 2026-09-11

**Authors:** Shuo Liu, Chao Bu, Tao Deng, Jinming Bai, Bin Luo, Mian Hou, Xiaoping Li, Jiansong Zhang, Wenyu Li, Zhihong Wang, Yanfei Feng, JiShan Wang, Dingqi Rao

**Affiliations:** 1 Kunming Natural History Museum of Zoology, Kunming Institute of Zoology, Chinese Academy of Sciences, Kunming, China Kunming Natural History Museum of Zoology, Kunming Institute of Zoology, Chinese Academy of Sciences Kunming China; 2 Key Laboratory of National Forestry and Grassland Administration on Biodiversity Conservation on the Qinghai-Xizang Plateau, Kunming Institute of Zoology, CAS, Kunming, China Key Laboratory of National Forestry and Grassland Administration on Biodiversity Conservation on the Qinghai-Xizang Plateau, Kunming Institute of Zoology, CAS Kunming China; 3 Yunnan Wenshan National Nature Reserve Management and Protection Bureau, Wenshan, China Yunnan Wenshan National Nature Reserve Management and Protection Bureau Wenshan China; 4 Yunnan Academy of Biodiversity, Southwest Forestry University, Kunming, China Yunnan Academy of Biodiversity, Southwest Forestry University Kunming China; 5 Southwest Survey and Planning Institute of National Forestry and Grassland Administration, Kunming, China Southwest Survey and Planning Institute of National Forestry and Grassland Administration Kunming China; 6 Yunnan Yongde Daxueshan National Nature Reserve Management and Protection Bureau, Yongde, China Yunnan Yongde Daxueshan National Nature Reserve Management and Protection Bureau Yongde China; 7 College of Continuing (Online) Education, Sichuan Normal University, Chengdu, China College of Continuing (Online) Education, Sichuan Normal University Chengdu China; 8 Wenshan City Management and Protection Sub-Bureau of Yunnan Wenshan National Nature Reserve, Wenshan, China Wenshan City Management and Protection Sub-Bureau of Yunnan Wenshan National Nature Reserve Wenshan China; 9 Kunming Institute of Zoology, Chinese Academy of Sciences, Kunming, China Kunming Institute of Zoology, Chinese Academy of Sciences Kunming China

**Keywords:** cryptic species, morphology, phylogeny, taxonomy, tree frog

## Abstract

**Background:**

*Zhangixalus puerensis* sensu lato was previously considered to be distributed in most parts of Yunnan and northern Vietnam. Some studies have implied that *Z. puerensis* sensu lato contains multiple independent species. However, the taxonomic status of *Z. puerensis* sensu lato remains unresolved.

**New information:**

Based on the sequences obtained from GenBank and our newly-generated sequences, we performed a taxonomic assessment of *Zhangixalus puerensis* sensu lato. Phylogenetic analysis revealed four distinct lineages within *Z. puerensis* sensu lato. We described two of the four lineages as two new species. The first new species from Wenshan Prefecture, Yunnan, China and Ha Giang Province, Vietnam, can be distinguished from its congeners by having a relatively small body size, head being wider than long, heels not meeting, tibiotarsal articulation reaching centre or anterior margin of tympanum, fifth toe being longer than third toe, nuptial pad being present on first finger and dorsal surface being green with more or less dark-edged brown spots. The second new species from Lincang City, Yunnan Province, China, can be distinguished from its congeners by having a relatively small body size, head being wider than long, heels not meeting, tibiotarsal articulation reaching posterior margin or centre of tympanum, fifth toe and third toe being approximately equal in length, dorsal surface green with no or a few or many dark-edged brown spots. In addition, the taxonomic status of populations previously considered to be *Z. puerensis* from north-western Yunnan of China and north-western Vietnam needs further research to determine.

## Introduction

The genus *Zhangixalus* Li, Jiang, Ren and Jiang, 2019 is a group of tree frogs mainly distributed in China and northern Indochina; it currently contains 46 species and new species have been continuously described in recent years (Frost 2026). Pan et al. (2025) considered that the actual diversity of this genus probably remains underestimated.

So far, 32 species of *Zhangixalus* have been recorded in China, with 12 species found in Yunnan Province (AmphibiaChina 2026). Amongst the 12 species of *Zhangixalus* distributed in Yunnan, *Z. puerensis* (He, 1999), which was previously referred as *Z. dugritei* (David, 1872), is the most widely recorded and is considered to be distributed in most parts of Yunnan, including north-western, western, south-western, central, southern and south-eastern Yunnan (Yang and Rao 2008, Fei et al. 2012, Yuan et al. 2022, AmphibiaChina 2026, Che and Lu 2026, Che and Yu 2026). In addition, *Z. puerensis* was also considered to be distributed in northern Vietnam (Li et al. 2012, Frost 2026).

During our field surveys in Yunnan Province, China, from 2020 to 2026, some specimens of *Zhangixalus puerensis* or *Z. cf. puerensis* were collected from multiple locations. Combining the sequences obtained from GenBank as well as our newly-generated sequences, we performed a taxonomic assessment of *Zhangixalus puerensis* sensu lato. *Zhangixalus puerensis* sensu lato formed four distinct lineages in the phylogenetic analysis, one of which represents Z*. puerensis* sensu stricto, while the other three represent three cryptic new species, with one from Lincang City, Yunnan, China, one from Lao Cai Province, Vietnam and one from Wenshan City, Yunnan, China and Ha Giang Province, Vietnam. In addition, we found that the two cryptic new species from Lincang, Yunnan, China and from Wenshan, Yunnan, China and Ha Giang, Vietnam, are morphologically different from Z*. puerensis* sensu stricto and from each other, while we did not yet obtain morphological data for the cryptic new species from Lao Cai, Vietnam. Therefore, we name and describe the two cryptic new species from Yunnan of China and Ha Giang of Vietnam herein. For the taxonomic status of the cryptic new species from Lao Cai of Vietnam, further research is needed to resolve it.

## Materials and methods

Specimens were collected by hand at night. After being photographed, euthanised and fixed, the specimens were stored in 75% ethanol and deposited at Kunming Natural History Museum of Zoology, Kunming Institute of Zoology, Chinese Academy of Sciences (KIZ).

Total genomic DNA was extracted from liver tissues. A fragment of the mitochondrial 16S rRNA gene was amplified and sequenced using the primers L2188 (5’–AAAGTGGGCCTAAAAGCAGCCA–3’) (Matsui and Panha 2006) and 16H1 (5’–CTCCGGTCTGAACTCAGATCACGTAGG–3’) (Hedges 1994). Amplification and sequencing were completed by Sangon Biotech Co., Ltd. (Shanghai, China). The newly-obtained sequences were deposited in GenBank and sequences of other *Zhangixalus* species and outgroups were obtained from GenBank (Table 1). *Theloderma albopunctatum* (Liu, 1962), *Rhacophorus rhodopus* (Liu & Hu, 1960) and *Leptomantis gauni* (Inger, 1966) were used as outgroups, according to Pan et al. (2025).

Sequences were aligned using MAFFT 7 (Katoh and Standley 2013) with default parameters. Average uncorrected p-distances between species were calculated in MEGA 12.0.10 (Kumar et al. 2024). The best substitution model was selected using the Bayesian information criterion in ModelFinder (Kalyaanamoorthy et al. 2017). Bayesian Inference and Maximum Likelihood analysis were used to construct phylogenetic trees. The technical computation methods for Bayesian Inference and Maximum Likelihood analyses were the same as those used in Liu et al. (2026).

Species delimitation was conducted using the method of Assemble Species by Automatic Partitioning (ASAP) and the model of simple distance. Lower ASAP-scores are considered better partitions according to Puillandre et al. (2021).

Measurements were taken using electronic digital calipers to the nearest 0.1 mm. Morphological terminology included: snout-vent length (SVL), head length (HL), head width (HW), snout length (SL), internarial distance (IND), interorbital distance (IOD), upper eyelid width (UEW), eye diameter (ED), distance between nostril and eye (DNE), tympanum diameter (TD), forearm and hand length (FHL), tibia length (TL), foot and tarsus length (TFL) and foot length (FL). The detailed measurement methods are the same as those in Pan et al. (2025) and the definition of webbing formula followed Myers and Duellman (1982).

## Taxon treatments

### Zhangixalus liui

Liu, Bu, Hou, Feng, Wang & Rao sp. nov.

AA4D7357-C263-5CA1-9BF2-260B09EAE0F6

A932307E-1A11-4A12-9416-EB615D98023C

#### Materials

- **Type status:** Holotype. **Occurrence:** catalogNumber: KIZ2025217; individualCount: 1; sex: male; lifeStage: adult; occurrenceID: BE5E9AF1-5BFC-5B5B-84D1-E24B82F1378F; **Location:** country: China; stateProvince: Yunnan; locality: Bozhu Town, Wenshan City, Wenshan Prefecture; verbatimElevation: 2540 m; verbatimCoordinates: 23°22'24''N 103°55'32''E; **Event:** eventRemarks: collected on 16 April 2025 by Chao Bu; **Record Level:** basisOfRecord: preserved specimen
- **Type status:** Paratype. **Occurrence:** catalogNumber: KIZ2025045; individualCount: 1; sex: male; lifeStage: adult; occurrenceID: F5DC67D4-FA08-5FF1-ACA3-20FC526891EE; **Location:** country: China; stateProvince: Yunnan; locality: Bozhu Town, Wenshan City, Wenshan Prefecture; verbatimElevation: 2540 m; verbatimCoordinates: 23°22'24''N 103°55'32''E; **Event:** eventRemarks: collected on 16 April 2025 by Chao Bu; **Record Level:** basisOfRecord: preserved specimen
- **Type status:** Paratype. **Occurrence:** catalogNumber: KIZ2025046; individualCount: 1; sex: male; lifeStage: adult; occurrenceID: 9EF81DAC-BEA3-51A3-8B00-1311EB0AFD1E; **Location:** country: China; stateProvince: Yunnan; locality: Bozhu Town, Wenshan City, Wenshan Prefecture; verbatimElevation: 2540 m; verbatimCoordinates: 23°22'24''N 103°55'32''E; **Event:** eventRemarks: collected on 16 April 2025 by Chao Bu; **Record Level:** basisOfRecord: preserved specimen
- **Type status:** Paratype. **Occurrence:** catalogNumber: KIZ2025047; individualCount: 1; sex: male; lifeStage: adult; occurrenceID: AF22699D-86C7-5CD8-A9CD-0442EAE1702D; **Location:** country: China; stateProvince: Yunnan; locality: Bozhu Town, Wenshan City, Wenshan Prefecture; verbatimElevation: 2540 m; verbatimCoordinates: 23°22'24''N 103°55'32''E; **Event:** eventRemarks: collected on 16 April 2025 by Chao Bu; **Record Level:** basisOfRecord: preserved specimen
- **Type status:** Paratype. **Occurrence:** catalogNumber: KIZ2025048; individualCount: 1; sex: male; lifeStage: adult; occurrenceID: 46414D9D-80FE-58DC-BEE9-EBF88E47DCCD; **Location:** country: China; stateProvince: Yunnan; locality: Bozhu Town, Wenshan City, Wenshan Prefecture; verbatimElevation: 2540 m; verbatimCoordinates: 23°22'24''N 103°55'32''E; **Event:** eventRemarks: collected on 16 April 2025 by Chao Bu; **Record Level:** basisOfRecord: preserved specimen
- **Type status:** Paratype. **Occurrence:** catalogNumber: KIZ2025049; individualCount: 1; sex: male; lifeStage: adult; occurrenceID: E68F4980-ABB8-599C-9792-A4F232E48219; **Location:** country: China; stateProvince: Yunnan; locality: Bozhu Town, Wenshan City, Wenshan Prefecture; verbatimElevation: 2540 m; verbatimCoordinates: 23°22'24''N 103°55'32''E; **Event:** eventRemarks: collected on 16 April 2025 by Chao Bu; **Record Level:** basisOfRecord: preserved specimen
- **Type status:** Paratype. **Occurrence:** catalogNumber: KIZ2025214; individualCount: 1; sex: male; lifeStage: adult; occurrenceID: 7F092868-7440-5B99-880E-C5303BC96F29; **Location:** country: China; stateProvince: Yunnan; locality: Bozhu Town, Wenshan City, Wenshan Prefecture; verbatimElevation: 2540 m; verbatimCoordinates: 23°22'24''N 103°55'32''E; **Event:** eventRemarks: collected on 16 April 2025 by Chao Bu; **Record Level:** basisOfRecord: preserved specimen
- **Type status:** Paratype. **Occurrence:** catalogNumber: KIZ2025215; individualCount: 1; sex: male; lifeStage: adult; occurrenceID: 82FD24B7-EE52-554D-A269-ADDD19AF2A7E; **Location:** country: China; stateProvince: Yunnan; locality: Bozhu Town, Wenshan City, Wenshan Prefecture; verbatimElevation: 2540 m; verbatimCoordinates: 23°22'24''N 103°55'32''E; **Event:** eventRemarks: collected on 16 April 2025 by Chao Bu; **Record Level:** basisOfRecord: preserved specimen
- **Type status:** Paratype. **Occurrence:** catalogNumber: KIZ2025216; individualCount: 1; sex: female; lifeStage: adult; occurrenceID: D0605239-7A4C-54A5-9A8F-ED4FDE090939; **Location:** country: China; stateProvince: Yunnan; locality: Bozhu Town, Wenshan City, Wenshan Prefecture; verbatimElevation: 2540 m; verbatimCoordinates: 23°22'24''N 103°55'32''E; **Event:** eventRemarks: collected on 16 April 2025 by Chao Bu; **Record Level:** basisOfRecord: preserved specimen

#### Etymology

The species epithet is in commemoration of the 50^th^ anniversary of the death of Prof. Chengzhao Liu, the founder of Chinese amphibian and reptile studies. According to the type locality of this species, we suggest “Bozhushan tree frog” as the English common name and “薄竹山树蛙” as the Chinese common name.

#### Diagnosis

*Zhangixalus liui* sp. nov. can be distinguished from its congeners by a combination of the following characters: body size relatively small, SVL 42.1‒46.4 mm in males; head wider than long; heels not meeting when legs positioned at right angle to body; tibiotarsal articulation reaching centre or anterior margin of tympanum when hind-limb stretched forwards; fifth toe longer than third toe; nuptial pad present on first finger; dorsal surface green with more or less dark-edged brown spots; large black patches on axilla and groin region; posterior surface of thigh mostly black.

#### Description of holotype

Adult male, body robust, size small (SVL 45.1 mm); head short (HL/SVL 0.36), head width slightly greater than head length (HW/HL 1.06); snout relatively short (SL/HL 0.49), rounded, tip slightly pointed; canthus rostralis distinct; nostril oval, slightly protuberant, closer to eye than to snout tip; internarial distance slightly greater than interorbital distance (IND/IOD 1.06); interorbital distance slightly greater than upper eyelid width (IOD/UEW 1.09); pineal spot absent; eye moderate (ED/HL 0.30), pupil horizontally oval; tympanum relatively small (TD/HL 0.18), distinct, rounded; supratympanic fold distinct; vomerine teeth distinct; choanae small, rounded; tongue relatively slender, notched posteriorly; single subgular vocal sac, sac openings large, slit-like.

Fore-limbs moderately long (FHL/SVL 0.53); relative length of fingers III > IV > II > I; tip of first finger slightly expanded, tips of second to fourth fingers significantly expanded into discs, all finger tips with circum-marginal grooves; nuptial pad present on first finger; webbing between fingers feebly developed, formula I2‒2⅓II2‒3III2⅓‒2IV; subarticular tubercles distinct, rounded, formula 1, 1, 2, 2, ones on first and second fingers and proximal ones on third and fourth fingers small, distal ones on third and fourth fingers large; supernumerary tubercles indistinct, small; inner metacarpal tubercle large, oval; outer metacarpal tubercle absent; dermal fringe along outer edge of forearm absent.

Hind-limbs short (TL/SVL 0.41; TFL/SVL 0.66), heels obviously not meeting when legs positioned at right angle to body; tibiotarsal articulation reaching anterior margin of tympanum when hind-limb stretched forwards; relative length of toes IV > V > III > II > I; tips of all toes expanded into discs with circum-marginal grooves; webbing between toes undeveloped, formula I1½‒2II1‒2⅓III1⅔‒2⅔IV2⅔‒1⅔V; subarticular tubercles distinct, small, rounded, formula 1, 1, 2, 3, 2; supernumerary tubercles absent; inner metatarsal tubercle oval; outer metatarsal tubercle absent; dermal fringe along outer edge of tarsus and tibia absent.

**Figure 1.**
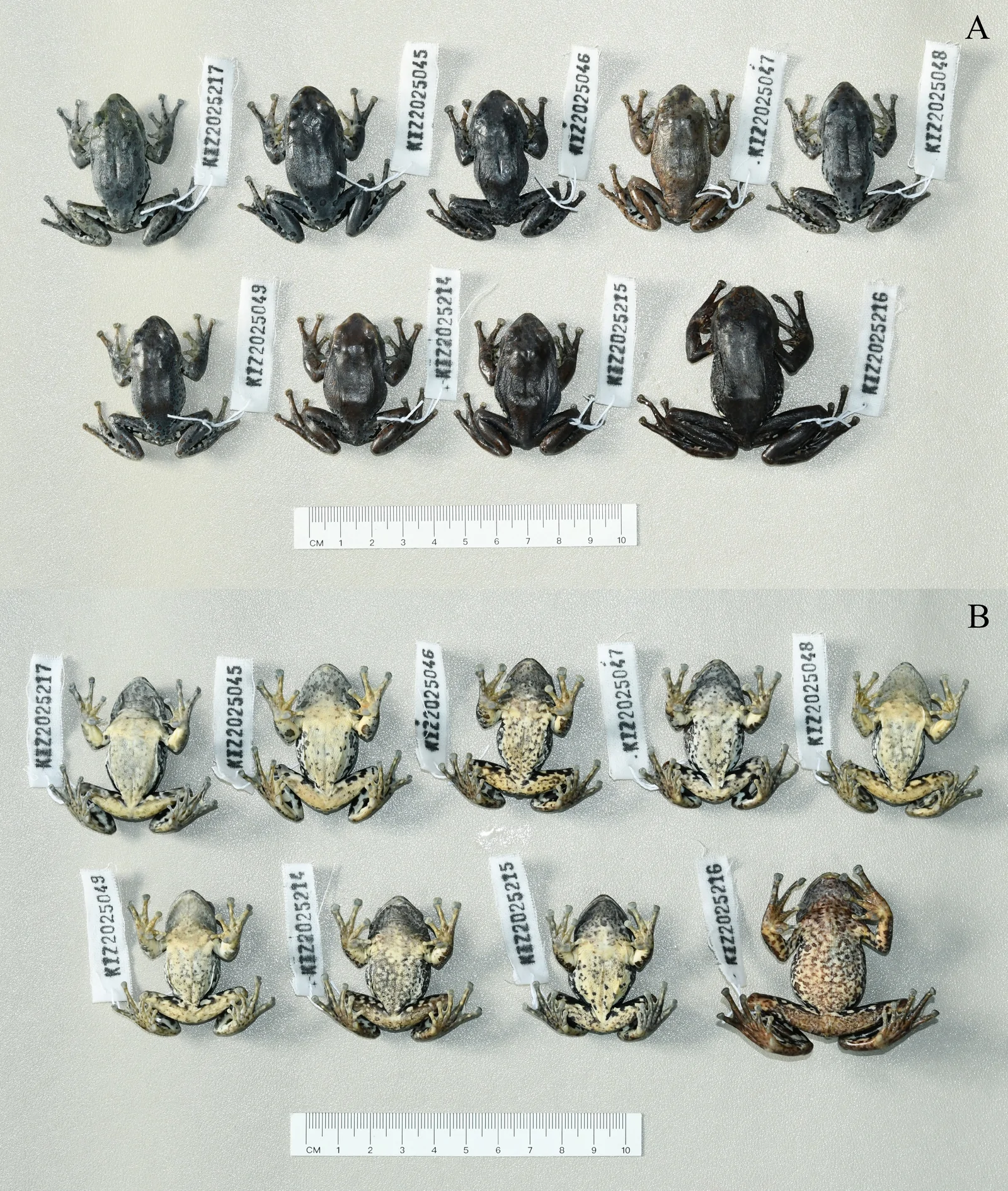
The type specimens of *Zhangixalus liui* sp. nov. in preservative. **A** dorsal view; **B** ventral view.

Dorsal surface of head, body and limbs slightly rough with dense small tubercles; ventral surface of head, body and thigh granular; ventral surface of fore-limbs, tibia and tarsus smooth (Fig. 1).

#### Colouration of holotype in life

**Figure 2.**
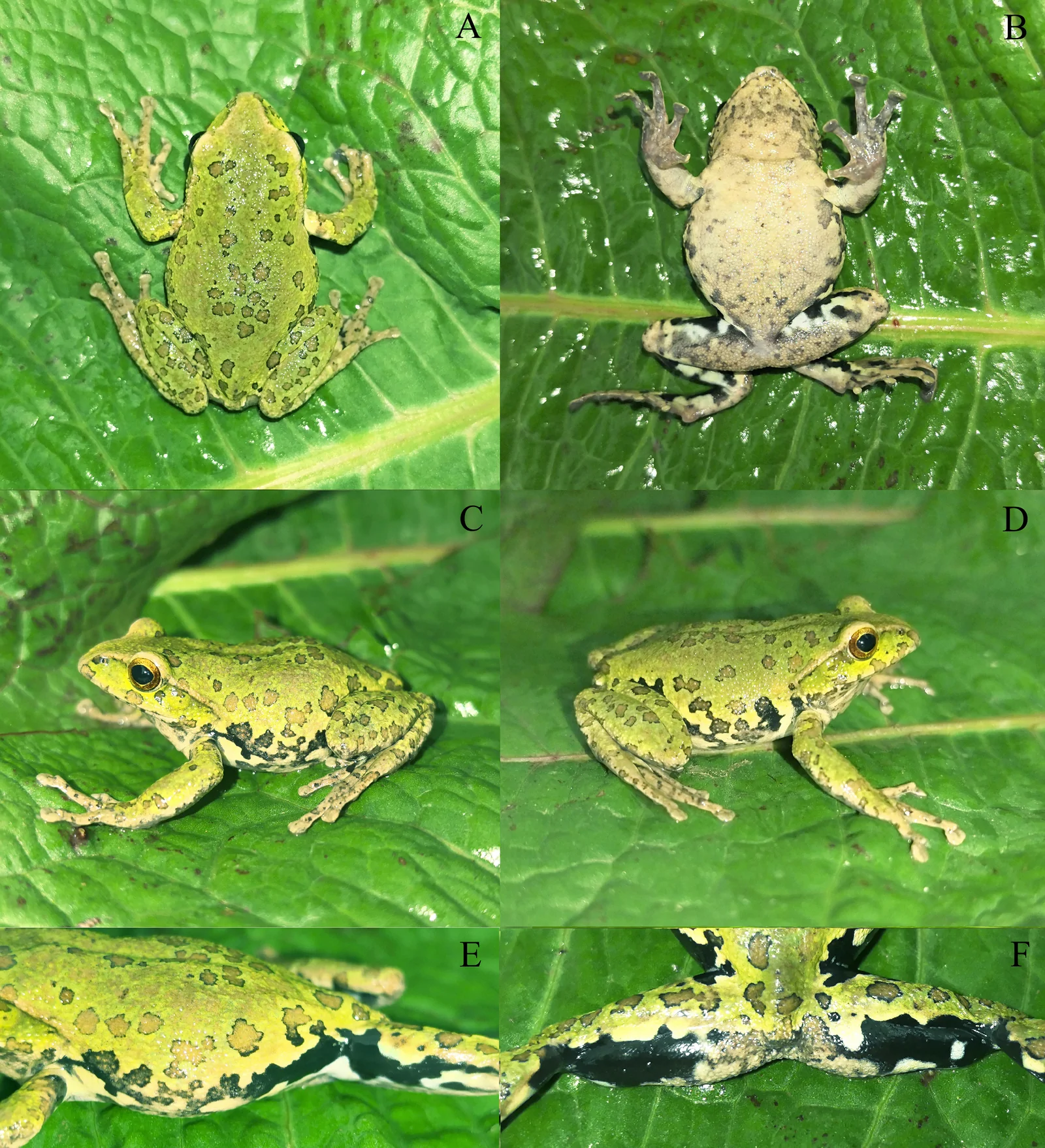
The holotype (KIZ2025217) of *Zhangixalus liui* sp. nov. in life. **A** dorsal view; **B** ventral view; **C** left view; **D** right view; **E** close-up view of the axilla, flank and groin region; **F** close-up view of the posterior surface of the thigh.

Dorsal surface green with some irregular-shaped dark-edged brown spots; flank yellowish-green to yellowish-white with some irregular-shaped brownish-black spots; large irregular-shaped black patches on axilla and groin region; posterior surface of thigh mostly black with a few white spots; ventral surface of head light yellow with many irregular-shaped dark patches or spots; ventral surface of body and thigh yellowish-white with some irregular-shaped dark spots; ventral surface of arm light yellowish-white; ventral surface of hand and foot grey; iris copper-coloured (Fig. 2).

#### Variations

**Figure 3.**
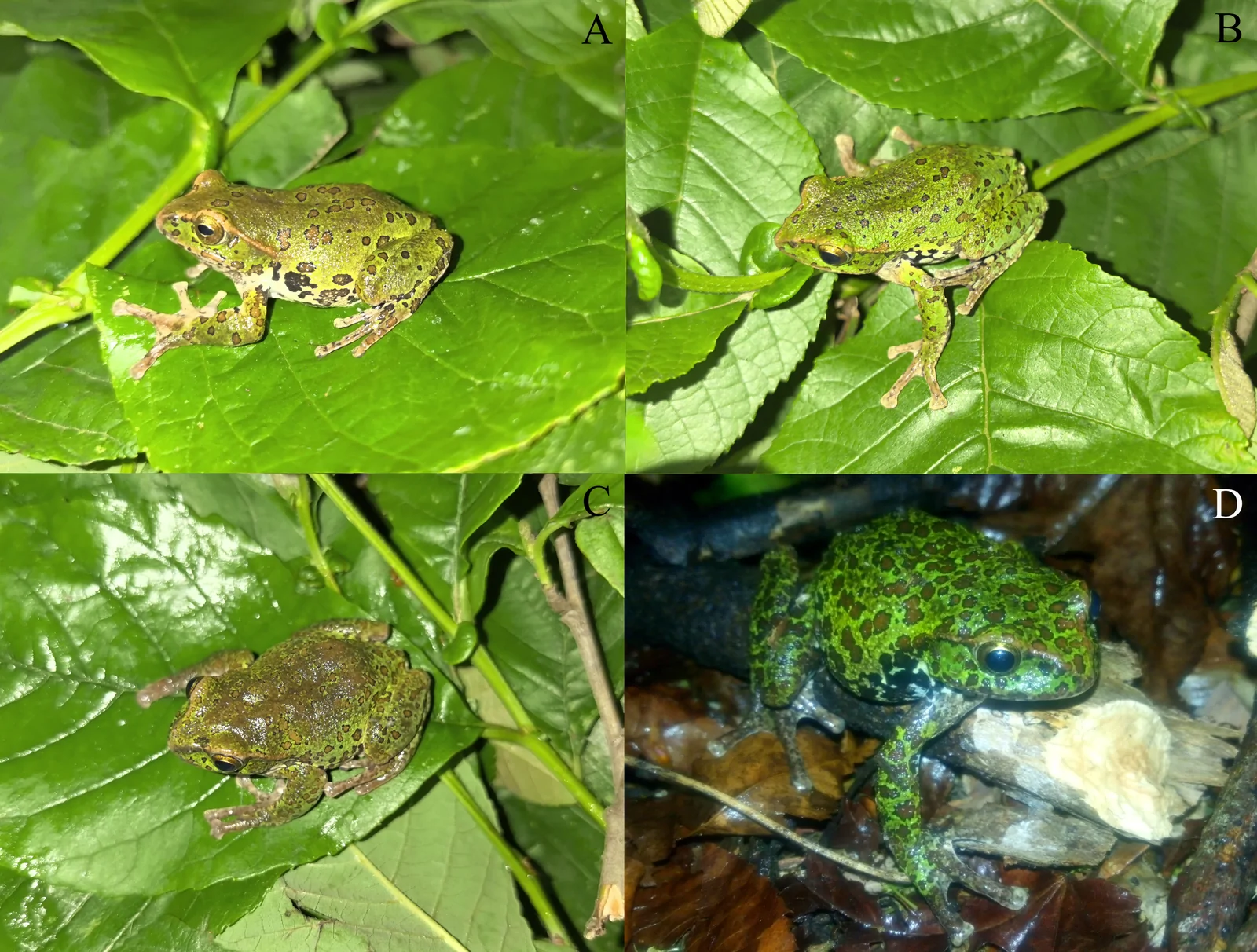
The paratypes of *Zhangixalus liui* sp. nov. in life. **A** KIZ2025045; **B** KIZ2025048; **C** KIZ2025049; **D** KIZ2025216.

The male paratypes are very similar in morphology to the holotype, except for slight differences in body size and the female paratype is obviously larger in size than the males (Table 2). In addition, there is no vocal sac and nuptial pad in the female. The colouration of this species varies not significantly; there are only some differences in the size and number of the brown spots on dorsal surface and no individuals with a pure green dorsal surface were observed (Fig. 3). However, this species has a very strong ability to change their body colour. When the surrounding environment changes or being threatened, the dorsal surface can turn brown with dark spots or almost completely brownish-black and the ventral surface can appear with more and darker spots.

#### Distribution and ecology

**Figure 4.**
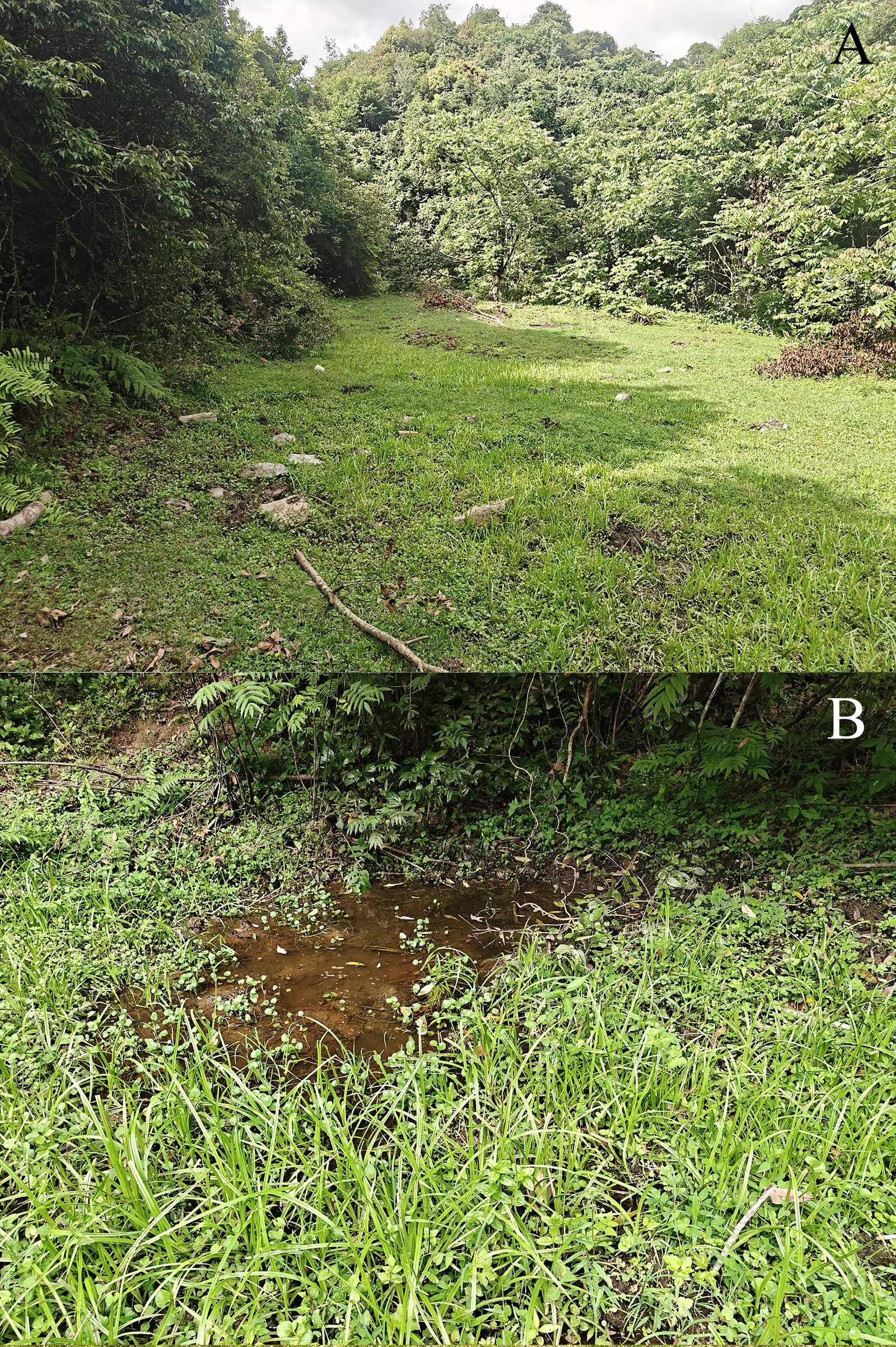
Distant view **(A)** and close-up view **(B)** of the habitat of *Zhangixalus liui* sp. nov. at the type locality.

This species is currently known from the type locality in Wenshan City, Wenshan Prefecture, Yunnan Province, China and Ha Giang Province, Vietnam. The type specimens of this species were discovered in holes in the mud or in grass beside a small pond. The pond is on a relatively flat grassland and there are a few similar ponds on the grassland. The grassland is surrounded by montane broadleaved forests (Fig. 4). Advertisement calls of this species were heard and foamy egg nests were found in holes in the mud, under rocks or in grass beside the pond in April and no advertisement calls were heard in July. The breeding period of this species is presumed to be during April to June.

#### Comparisons

*Zhangixalus liui* sp. nov. can be easily distinguished from all other species of the genus *Zhangixalus*, except for *Z. dugritei*, *Z. hongchibaensis* (Li, Liu, Chen, Wu, Murphy, Zhao, Wang & Zhang, 2012), *Z. hui* (Liu, 1945), *Z. puerensis* and *Z. wui* (Li, Liu, Chen, Wu, Murphy, Zhao, Wang & Zhang, 2012), by the body size and the presence of more or less brown spots with dark edges on the green dorsal surface.

*Zhangixalus liui* sp. nov. can be distinguished from *Z. dugritei* by the distance between the nostril and the snout tip being obviously greater than that between the nostril and the eye (vs. the distance between the nostril and the snout tip being approximately equal to that between the nostril and the eye), the webbing on the outer side of the third finger not reaching the second subarticular tubercle (vs. the webbing on the outer side of the third finger reaching the second subarticular tubercle), the fifth toe being longer than the third toe (vs. the fifth toe and the third toe being equal in length) and the nuptial pad being present on the first finger (vs. the nuptial pad being present on the first and second fingers).

*Zhangixalus liui* sp. nov. can be distinguished from *Z. hongchibaensis* by having a relatively smaller body size in males (42.1‒46.4 mm vs. 46.5–49.7 mm), the tibiotarsal articulation reaching the centre or the anterior margin of the tympanum when the hind-limb stretched forwards (vs. the tibiotarsal articulation not reaching the tympanum) and having a relatively longer tibia in males (TL/SVL 0.40–0.42 vs. 0.36–0.39).

*Zhangixalus liui* sp. nov. can be distinguished from *Z. hui* by the internarial distance being greater than the interorbital distance (vs. the internarial distance being equal to the interorbital distance), the tibiotarsal articulation reaching the centre or the anterior margin of the tympanum when the hind-limb stretched forwards (vs. the tibiotarsal articulation reaching the base of the fore-limb), the nuptial pad being present on the first finger (vs. the nuptial pad being present on the first and second fingers) and the absence of the outer metatarsal tubercle (vs. the presence of the outer metatarsal tubercle).

*Zhangixalus liui* sp. nov. can be distinguished from *Z. puerensis* by having a relatively larger body size in males (42.1‒46.4 mm vs. 35.5–41.1 mm), the tibiotarsal articulation reaching the centre or the anterior margin of the tympanum when the hind-limb stretched forwards (vs. the tibiotarsal articulation obviously not reaching the tympanum), the heels obviously not meeting when legs positioned at right angles to body (vs. the heels meeting each other), the fifth toe being longer than the third toe (vs. the fifth toe and the third toe being equal in length) and the nuptial pad being present on the first finger (vs. the nuptial pad being present on the first and second fingers).

*Zhangixalus liui* sp. nov. can be distinguished from *Z. wui* by having a relatively larger body size in males (42.1‒46.4 mm vs. 35.2–38.2 mm), the tibiotarsal articulation reaching the centre or the anterior margin of the tympanum when the hind-limb stretched forwards (vs. the tibiotarsal articulation not reaching the tympanum) and the absence of the tarsal fold (vs. the presence of a distinct tarsal fold).

### Zhangixalus orlovi

Liu, Wang, Bai, Deng, Hou & Rao sp. nov.

E7724486-98D3-5019-8751-055448810813

E251D1E9-B6D5-4FEA-BD7B-FABB8A1506BD

#### Materials

- **Type status:** Holotype. **Occurrence:** catalogNumber: KIZ2026024; individualCount: 1; sex: male; lifeStage: adult; occurrenceID: 9B4109E2-F481-53B2-BD41-47CC5107CF64; **Location:** country: China; stateProvince: Yunnan; locality: Daxueshan Township, Yongde County, Lincang City; verbatimElevation: 2940 m; verbatimCoordinates: 24°3'40''N 99°36'40''E; **Event:** eventRemarks: collected on 10 May 2026 by Jinming Bai; **Record Level:** basisOfRecord: preserved specime
- **Type status:** Paratype. **Occurrence:** catalogNumber: KIZ2026015; individualCount: 1; sex: male; lifeStage: adult; occurrenceID: 2DE7EF1F-FCD5-50CA-B7C3-63334573EF7B; **Location:** country: China; stateProvince: Yunnan; locality: Daxueshan Township, Yongde County, Lincang City; verbatimElevation: 2940 m; verbatimCoordinates: 24°3'40''N 99°36'40''E; **Event:** eventRemarks: collected on 10 May 2026 by Jinming Bai; **Record Level:** basisOfRecord: preserved specime
- **Type status:** Paratype. **Occurrence:** catalogNumber: KIZ2026016; individualCount: 1; sex: male; lifeStage: adult; occurrenceID: ACEE7EA1-57D5-5B97-986D-8D8372196C76; **Location:** country: China; stateProvince: Yunnan; locality: Daxueshan Township, Yongde County, Lincang City; verbatimElevation: 2940 m; verbatimCoordinates: 24°3'40''N 99°36'40''E; **Event:** eventRemarks: collected on 10 May 2026 by Jinming Bai; **Record Level:** basisOfRecord: preserved specime
- **Type status:** Paratype. **Occurrence:** catalogNumber: KIZ2026017; individualCount: 1; sex: male; lifeStage: adult; occurrenceID: 6F88714A-C2F9-5706-A2B5-D315DE731A14; **Location:** country: China; stateProvince: Yunnan; locality: Daxueshan Township, Yongde County, Lincang City; verbatimElevation: 2940 m; verbatimCoordinates: 24°3'40''N 99°36'40''E; **Event:** eventRemarks: collected on 10 May 2026 by Jinming Bai; **Record Level:** basisOfRecord: preserved specime
- **Type status:** Paratype. **Occurrence:** catalogNumber: KIZ2026018; individualCount: 1; sex: male; lifeStage: adult; occurrenceID: 1F5CE9CD-6180-5F16-A32B-EBE15E80FAEC; **Location:** country: China; stateProvince: Yunnan; locality: Daxueshan Township, Yongde County, Lincang City; verbatimElevation: 2940 m; verbatimCoordinates: 24°3'40''N 99°36'40''E; **Event:** eventRemarks: collected on 10 May 2026 by Jinming Bai; **Record Level:** basisOfRecord: preserved specime
- **Type status:** Paratype. **Occurrence:** catalogNumber: KIZ2026019; individualCount: 1; sex: male; lifeStage: adult; occurrenceID: AC708E5C-9320-5E41-87F2-837DA65E08CE; **Location:** country: China; stateProvince: Yunnan; locality: Daxueshan Township, Yongde County, Lincang City; verbatimElevation: 2940 m; verbatimCoordinates: 24°3'40''N 99°36'40''E; **Event:** eventRemarks: collected on 10 May 2026 by Jinming Bai; **Record Level:** basisOfRecord: preserved specime
- **Type status:** Paratype. **Occurrence:** catalogNumber: KIZ2026020; individualCount: 1; sex: female; lifeStage: adult; occurrenceID: 7B25C320-5232-54BF-B464-359CBA8E8D80; **Location:** country: China; stateProvince: Yunnan; locality: Daxueshan Township, Yongde County, Lincang City; verbatimElevation: 2940 m; verbatimCoordinates: 24°3'40''N 99°36'40''E; **Event:** eventRemarks: collected on 10 May 2026 by Jinming Bai; **Record Level:** basisOfRecord: preserved specime
- **Type status:** Paratype. **Occurrence:** catalogNumber: KIZ2026021; individualCount: 1; sex: female; lifeStage: adult; occurrenceID: 08238A7A-58D4-5516-BFF1-181D9A7249BE; **Location:** country: China; stateProvince: Yunnan; locality: Daxueshan Township, Yongde County, Lincang City; verbatimElevation: 2940 m; verbatimCoordinates: 24°3'40''N 99°36'40''E; **Event:** eventRemarks: collected on 10 May 2026 by Jinming Bai; **Record Level:** basisOfRecord: preserved specime
- **Type status:** Paratype. **Occurrence:** catalogNumber: KIZ2026022; individualCount: 1; sex: male; lifeStage: adult; occurrenceID: A558A2EE-6EAE-54C1-ACA3-178786079185; **Location:** country: China; stateProvince: Yunnan; locality: Daxueshan Township, Yongde County, Lincang City; verbatimElevation: 2940 m; verbatimCoordinates: 24°3'40''N 99°36'40''E; **Event:** eventRemarks: collected on 10 May 2026 by Jinming Bai; **Record Level:** basisOfRecord: preserved specime
- **Type status:** Paratype. **Occurrence:** catalogNumber: KIZ2026023; individualCount: 1; sex: male; lifeStage: adult; occurrenceID: 87CBA786-31DD-5D2D-8954-F4E3EE8673C2; **Location:** country: China; stateProvince: Yunnan; locality: Daxueshan Township, Yongde County, Lincang City; verbatimElevation: 2940 m; verbatimCoordinates: 24°3'40''N 99°36'40''E; **Event:** eventRemarks: collected on 10 May 2026 by Jinming Bai; **Record Level:** basisOfRecord: preserved specime

#### Etymology

The specific epithet is in commemoration of the renowned Russian herpetological taxonomist Prof. Dr Nikolai Liutsianovich Orlov, who recently passed away. According to the type locality of this species, we suggest “Daxueshan tree frog” as the English common name and “大雪山树蛙” as the Chinese common name.

#### Diagnosis

*Zhangixalus orlovi* sp. nov. can be distinguished from its congeners by a combination of the following characters: body size small, SVL 34.0‒41.3 mm in males; head wider than long; heels not meeting when legs positioned at right angles to body; tibiotarsal articulation reaching posterior margin or centre of tympanum when hind-limb stretched forwards; fifth toe and third toe approximately equal in length; dorsal surface green with no or a few or many dark-edged brown spots; small black patches or streak on axilla and groin region; posterior surface of thigh black with many white spots.

#### Description of holotype

Adult male, body robust, size small (SVL 41.3 mm); head short (HL/SVL 0.34), head width slightly greater than head length (HW/HL 1.04); snout relatively short (SL/HL 0.47), rounded, tip slightly pointed; canthus rostralis distinct; nostril oval, slightly protuberant, closer to eye than to snout tip; internarial distance greater than interorbital distance (IND/IOD 1.12); interorbital distance greater than upper eyelid width (IOD/UEW 1.24); pineal spot absent; eye moderate (ED/HL 0.32), pupil horizontally oval; tympanum relatively small (TD/HL 0.17), distinct, rounded; supratympanic fold distinct; vomerine teeth distinct; choanae small, slightly oval; tongue relatively slender, notched posteriorly; single subgular vocal sac, sac openings large, slit-like.

Fore-limbs moderately long (FHL/SVL 0.52); relative length of fingers III > IV > II > I; tip of first finger slightly expanded, tips of second to fourth fingers significantly expanded into discs, all finger tips with circum-marginal grooves; nuptial pad present on first and second fingers; webbing between fingers feebly developed, formula I2½‒2½II2‒3III2⅓‒2IV; subarticular tubercles distinct, rounded, formula 1, 1, 2, 2, ones on first and second fingers and proximal ones on third and fourth fingers small, distal ones on third and fourth fingers large; supernumerary tubercles indistinct, small; inner metacarpal tubercle large, oval; outer metacarpal tubercle absent; dermal fringe along outer edge of forearm absent.

Hind-limbs short (TL/SVL 0.40; TFL/SVL 0.65), heels obviously not meeting when legs positioned at right angles to body; tibiotarsal articulation reaching centre of tympanum when hind-limb stretched forwards; relative length of toes IV > V ≈ III > II > I; tips of all toes expanded into discs with circum-marginal grooves; webbing between toes undeveloped, formula I1⅔‒2II1½‒2½III2‒3IV2⅔‒1⅔V; subarticular tubercles distinct, small, rounded, formula 1, 1, 2, 3, 2; supernumerary tubercles absent; inner metatarsal tubercle oval; outer metatarsal tubercle absent; dermal fringe along outer edge of tarsus and tibia absent.

**Figure 5.**
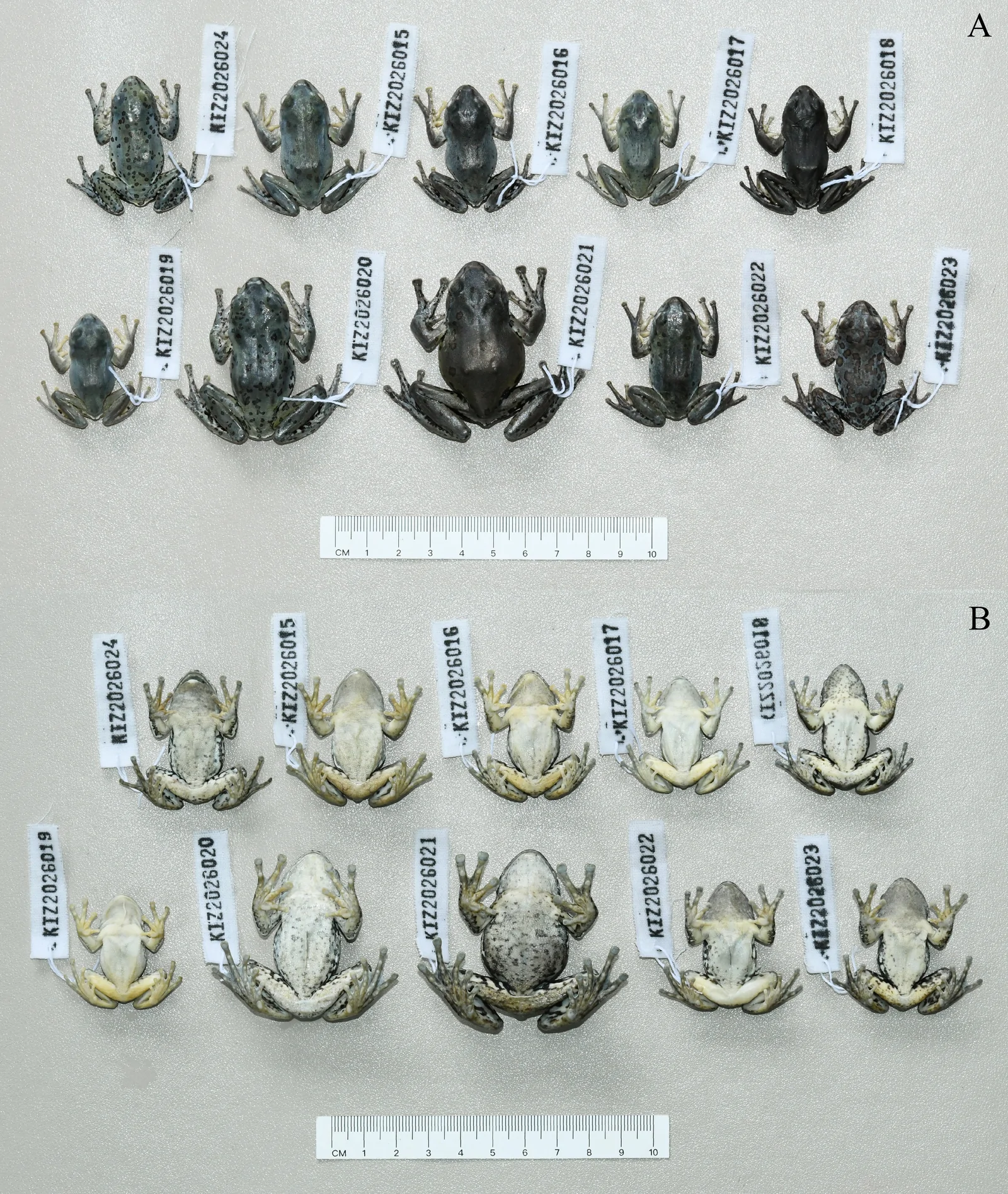
The type specimens of *Zhangixalus orlovi* sp. nov. in preservative. **A** dorsal view; **B** ventral view.

Dorsal surface of head, body and limbs relatively smooth with dense tiny tubercles; ventral surface of head slightly granular; ventral surface of body granular; ventral surface of thigh granular with some protruding round tubercles; ventral surface of fore-limbs, tibia and tarsus smooth (Fig. 5).

#### Colouration of holotype in life

**Figure 6.**
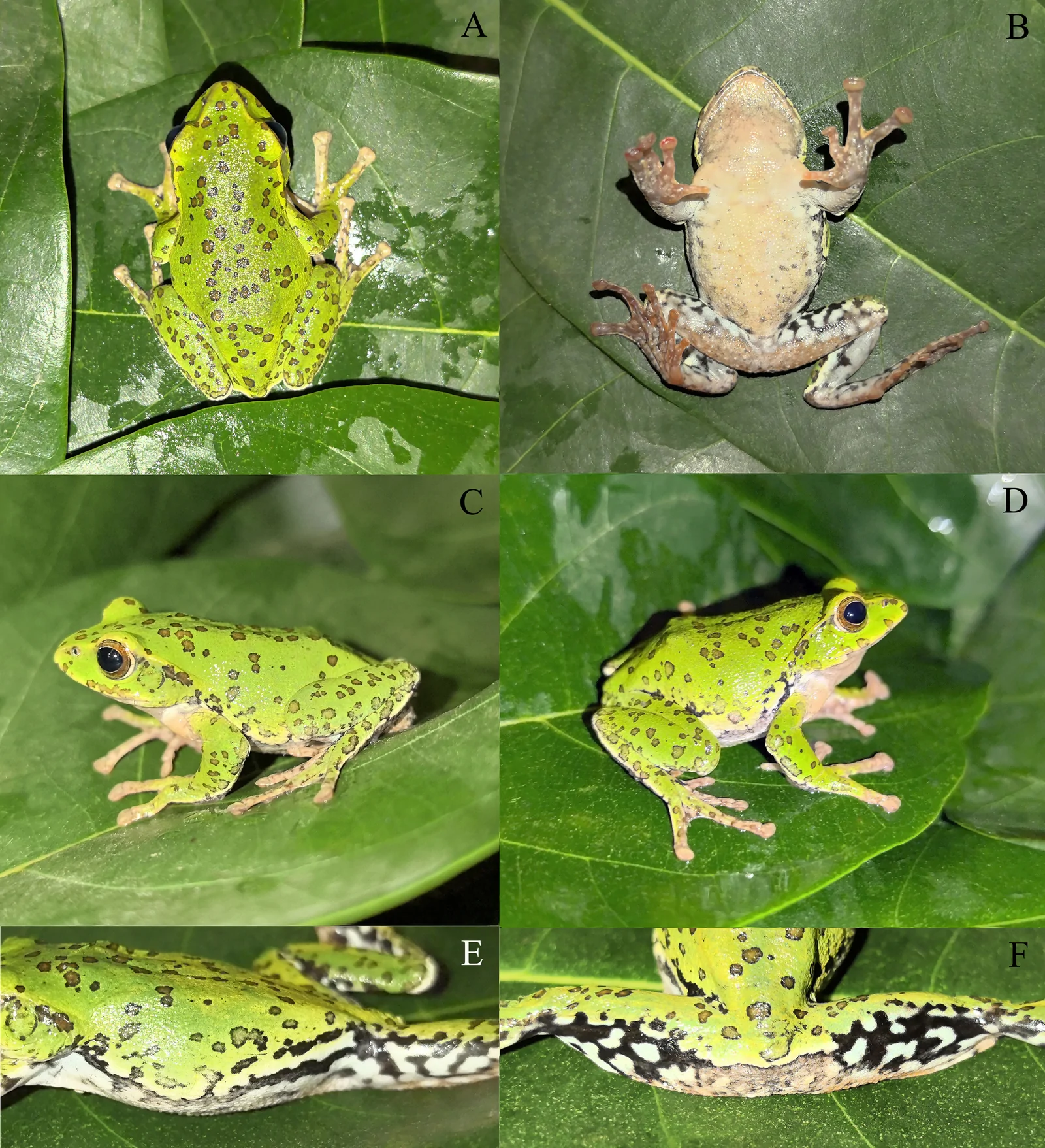
The holotype (KIZ2026024) of *Zhangixalus orlovi* sp. nov. in life. **A** dorsal view; **B** ventral view; **C** left view; **D** right view; **E** close-up view of the axilla, flank and groin region; **F** close-up view of the posterior surface of the thigh.

Dorsal surface green with some irregular-shaped dark-edged brown spots; flank yellowish-green to yellowish-white with some irregular-shaped greyish-black spots; small irregular-shaped black patches or streak on axilla and groin region; posterior surface of thigh black with many white spots; ventral surface of head and body yellowish-white with some small dark spots; ventral surface of thigh reddish-white; ventral surface of arm white; ventral surface of hand and foot light orange red; iris copper-coloured (Fig. 6).

#### Variations

**Figure 7.**
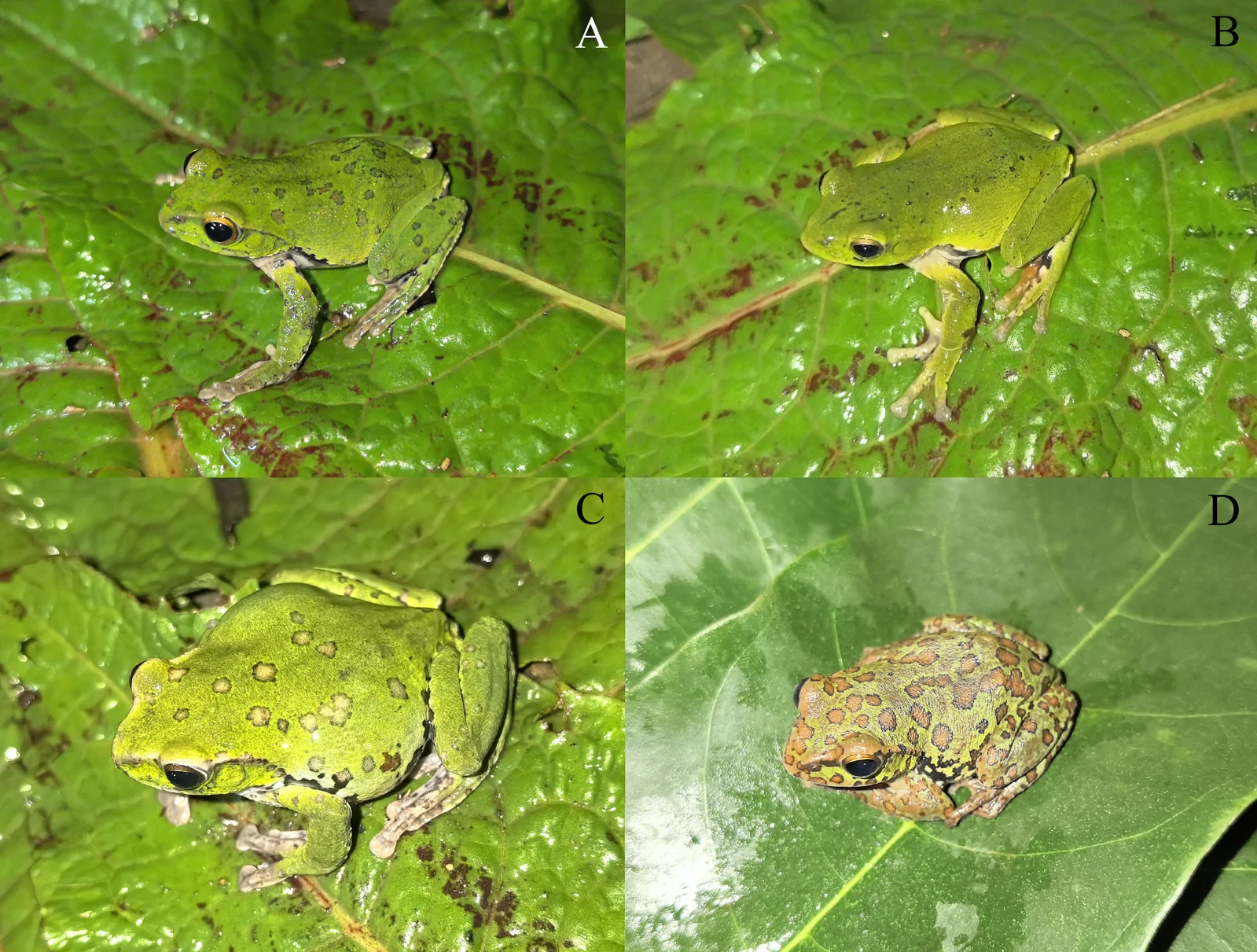
The paratypes of *Zhangixalus orlovi* sp. nov. in life. **A** KIZ2026015; **B** KIZ2026018; **C** KIZ2026021; **D** KIZ2026023.

The male paratypes are very similar in morphology to the holotype, except for slight differences in body size and the female paratypes are obviously larger in size than the males (Table 3). In addition, there is no vocal sac and nuptial pad in the females. The colouration of this species varies significantly, some individuals having a few small or large spots on the dorsal surface and some individuals having many small or large spots on the dorsal surface; in addition, some individuals have no spots on the dorsal surface, which appears pure green (Fig. 7). Similarly, this species also has a very strong ability to change their body colour. When the surrounding environment changes or being threatened, the dorsal surface can turn brown to brownish-black and the ventral surface can appear with more and darker spots.

#### Distribution and ecology

**Figure 8.**
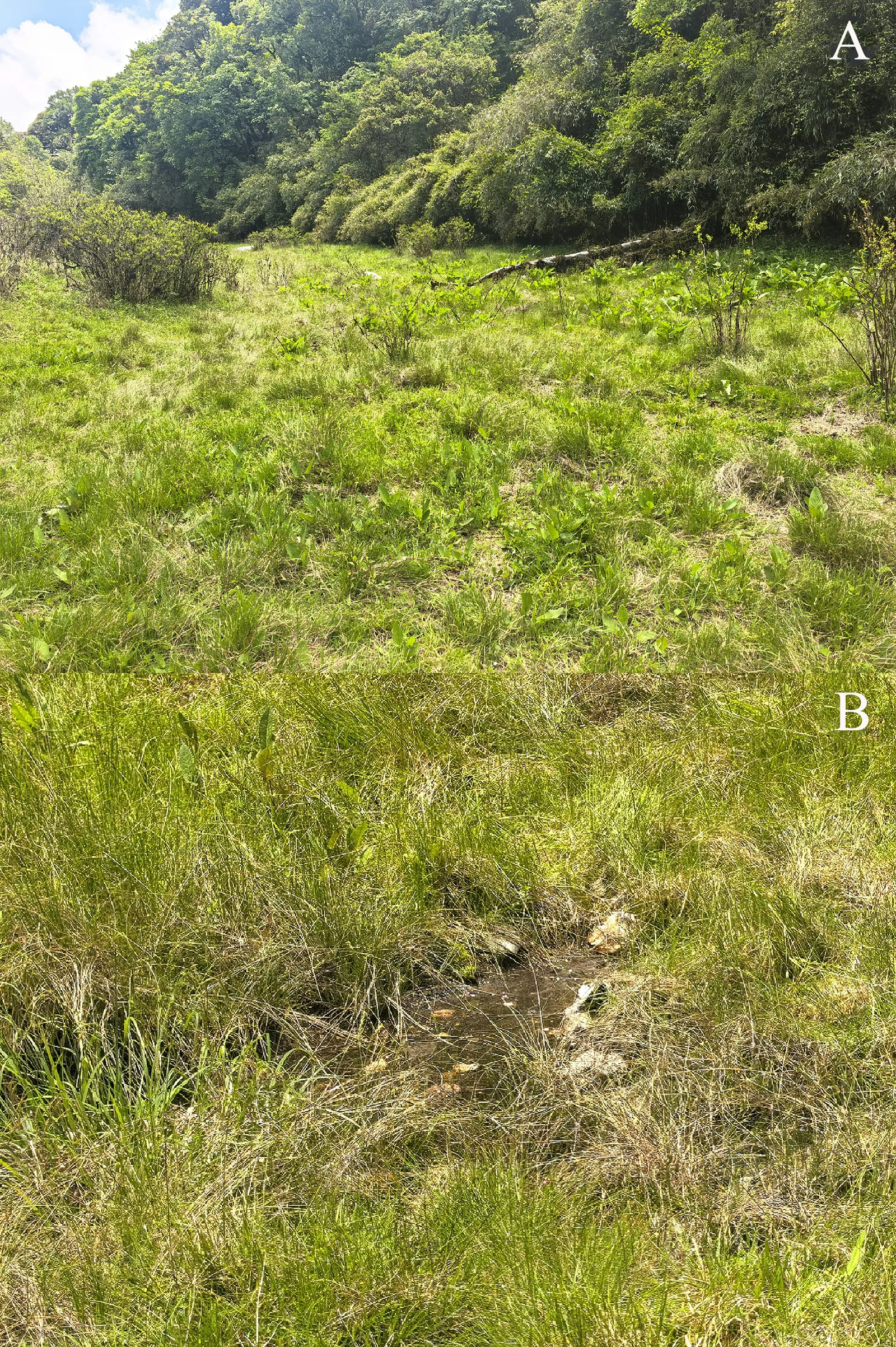
Distant view **(A)** and close-up view **(B)** of the habitat of *Zhangixalus orlovi* sp. nov. at the type locality.

This species is currently known only from the type locality in Yongde County, Lincang City, Yunnan Province, China. The type specimens of this species were discovered in holes in the mud or under rocks beside a small pond. The pond is on a relatively flat grassland and there are many similar ponds on the grassland. The grassland is surrounded by montane broadleaved forests (Fig. 8). Advertisement calls of this species were heard and foamy egg nests were found in holes in the mud, under rocks or in grass beside the pond in April and May and no advertisement calls were heard in July. The breeding period of this species is presumed to be during April to June.

#### Comparisons

*Zhangixalus orlovi* sp. nov. can be easily distinguished from all other species of the genus *Zhangixalus*, except for *Z. dugritei*, *Z. hongchibaensis* (Li, Liu, Chen, Wu, Murphy, Zhao, Wang & Zhang, 2012), *Z. hui* (Liu, 1945), *Z. puerensis*, *Z. wui* (Li, Liu, Chen, Wu, Murphy, Zhao, Wang & Zhang, 2012) and *Zhangixalus liui* sp. nov. by the body size and the presence of more or less brown spots with dark edges on the green dorsal surface.

*Zhangixalus orlovi* sp. nov. can be distinguished from *Z. dugritei* by the distance between the nostril and the snout tip being obviously greater than that between the nostril and the eye (vs. the distance between the nostril and the snout tip being approximately equal to that between the nostril and the eye), the upper eyelid width being greater than three-quarters of the interorbital distance (vs. the upper eyelid width being approximately two-thirds of the interorbital distance) and the webbing on the outer side of the third finger not reaching the second subarticular tubercle (vs. the webbing on the outer side of the third finger reaching the second subarticular tubercle).

*Zhangixalus orlovi* sp. nov. can be distinguished from *Z. hongchibaensis* by having a relatively smaller body size in males (34.0‒41.3 mm vs. 46.5–49.7 mm), the tibiotarsal articulation reaching the posterior margin or the centre of the tympanum when the hind-limb stretched forwards (vs. the tibiotarsal articulation not reaching the tympanum), having a relatively longer tibia in males (TL/SVL 0.40–0.45 vs. 0.36–0.39) and the nuptial pad being present on the first and second fingers (vs. the nuptial pad being present on the first finger).

*Zhangixalus orlovi* sp. nov. can be distinguished from *Z. hui* by the internarial distance being greater than the interorbital distance (vs. the internarial distance being equal to the interorbital distance), the tibiotarsal articulation reaching the posterior margin or the centre of the tympanum when the hind-limb stretched forwards (vs. the tibiotarsal articulation reaching the base of the fore-limb) and the absence of the outer metatarsal tubercle (vs. the presence of the outer metatarsal tubercle).

*Zhangixalus orlovi* sp. nov. can be distinguished from *Z. puerensis* by the tibiotarsal articulation reaching the posterior margin or the centre of the tympanum when the hind-limb stretched forwards (vs. the tibiotarsal articulation obviously not reaching the tympanum), the heels obviously not meeting when legs positioned at right angles to body (vs. the heels meeting each other) and the forearm and hand length being great than half of the snout-vent length (vs. the forearm and hand length being slightly smaller than or equal to half of the snout-vent length).

*Zhangixalus orlovi* sp. nov. can be distinguished from *Z. wui* by the tibiotarsal articulation reaching the posterior margin or the centre of the tympanum when the hind-limb stretched forwards (vs. the tibiotarsal articulation not reaching the tympanum), the absence of the tarsal fold (vs. the presence of a distinct tarsal fold) and the nuptial pad being present on the first and second fingers (vs. the nuptial pad being present on the first finger).

*Zhangixalus orlovi* sp. nov. can be distinguished from *Zhangixalus liui* sp. nov. by having a relatively smaller body size in males (34.0‒41.3 mm vs. 42.1‒46.4 mm), the fifth toe and the third toe being approximately equal in length (vs. the fifth toe being longer than the third toe), the nuptial pad being present on the first and second fingers (vs. the nuptial pad being present on the first finger), axilla and groin region and posterior surface of the thigh with small, scattered black patches or streaks (vs. axilla and groin region and posterior surface of the thigh with large, continuous black patches or streaks) and no or a few small dark patches being present on ventral surface (vs. many small to large dark patches being present on ventral surface).

## Analysis

**Figure 9.**
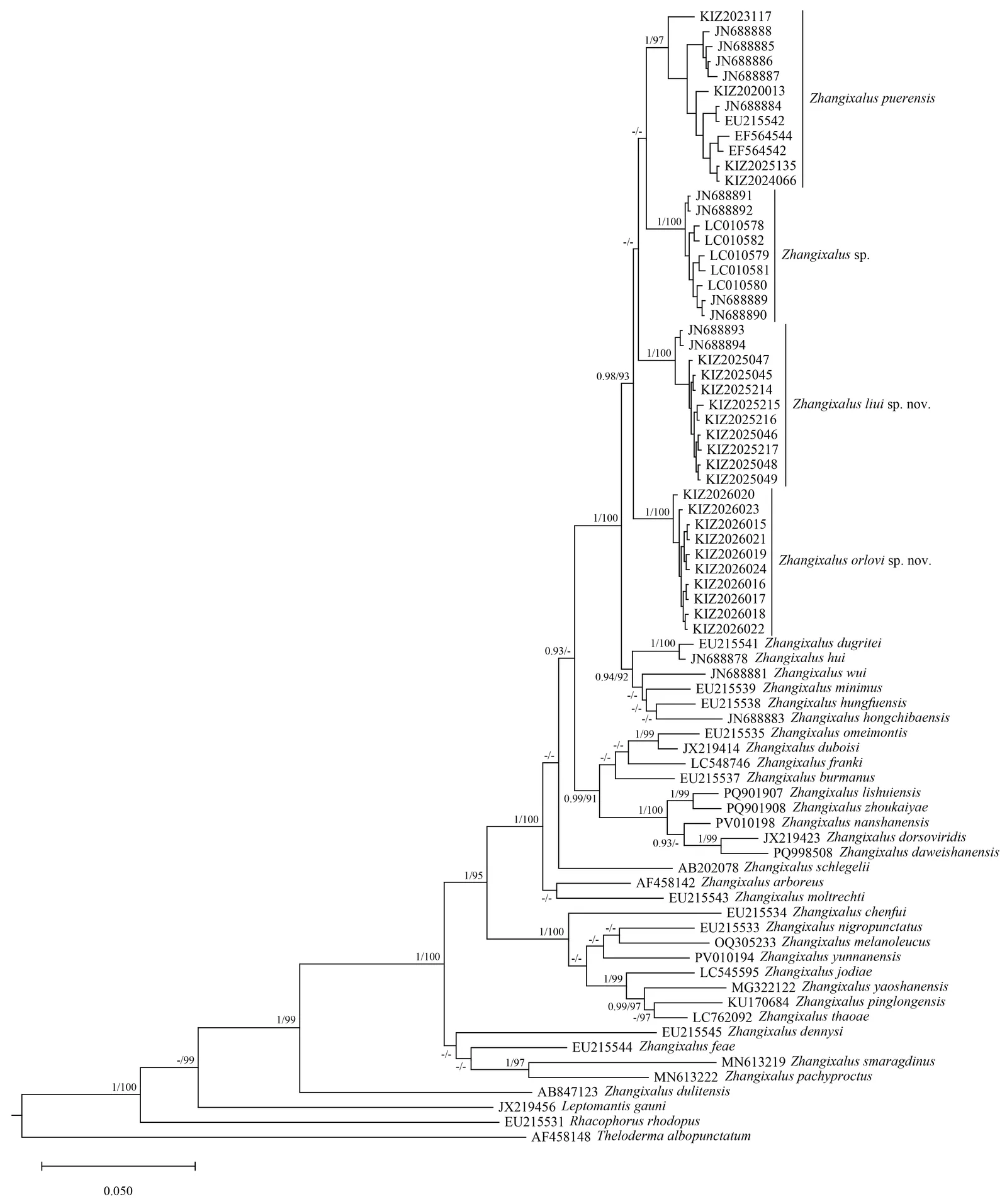
Bayesian phylogram of *Zhangixalus* inferred from 16S gene sequences. Numbers before and after the slash are Bayesian posterior probabilities and Maximum Likelihood bootstrap values (only values above 0.9/90 are shown), respectively.

The two phylogenetic analyses yielded essentially consistent results. *Zhangixalus puerensis* sensu lato were divided into four distinct clades, but the phylogenetic relationships amongst the clades have not been resolved due to very low node support rates (Fig. 9). The first clade was composed of samples from Pu'er City, Yunnan Province, China, which is the type locality of *Z. puerensis* and samples from Baoshan City, Dali Prefecture, Yuxi City and Honghe Prefecture of Yunnan Province, China, representing Z*. puerensis* sensu stricto; the second clade was composed of samples from Sa Pa District, Lao Cai Province, Vietnam; the third clade was composed of samples from Wenshan Prefecture of Yunnan Province, China and Ha Giang Province of Vietnam; and the fourth clade was composed of samples from Lincang City of Yunnan Province, China. The genetic distances amongst the four clades ranged from 2.2% to 2.6% (Suppl. material 1), obviously greater than those between *Z. dugritei* and *Z. hui* (0.5%), between *Z. duboisi* and *Z. meimontis* (1.6%) and between *Z. lishuiensis* and *Z. zhoukaiyae* (1.5%) and approximately equivalent to those between *Z. lishuiensis* and *Z. nanshanensis* (2.4%), between *Z. daweishanensis* and *Z. dorsoviridis* (2.5%), between *Z. hungfuensis* and *Z. minimus* (2.6%), between *Z. dugritei* and *Z. minimus* (2.7%) and between *Z. hui* and *Z. minimus* (2.7%).

**Figure 10.**
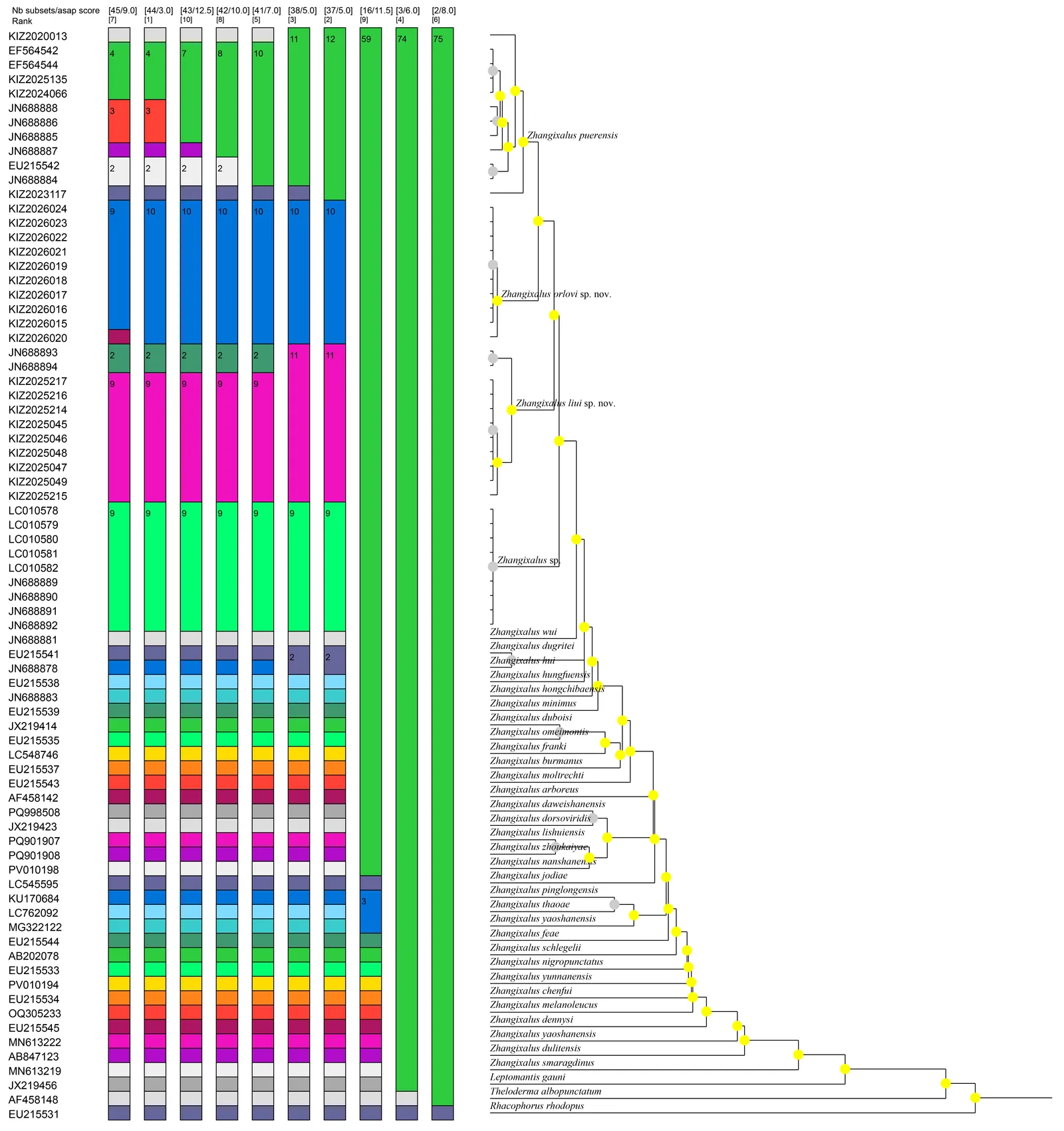
The ASAP species delimitation, based on 16S rRNA sequences.

The partition with the lowest score (3.0) obtained by the ASAP analysis assigned the samples from Wenshan Prefecture and Lincang City of Yunnan Province, China, as well as Sa Pa District of Lao Cai Province, Vietnam, as three distinct species. Besides, it assigned the samples of *Zhangixalus puerensis* from different localities as different species and also assigned the samples from Ha Giang Province of Vietnam as a distinct species. The partition with the second lowest score (5.0) corresponded exactly to the result of the phylogenetic analysis, except that it assigned *Z. dugritei* and *Z. hui* as one species (Fig. 10). Therefore, the ASAP analysis supports the samples from Wenshan Prefecture and Lincang City of Yunnan Province, China, as well as Sa Pa District of Lao Cai Province, Vietnam, as three species distinct from Z*. puerensis* sensu stricto.

## Discussion

**Figure 11.**
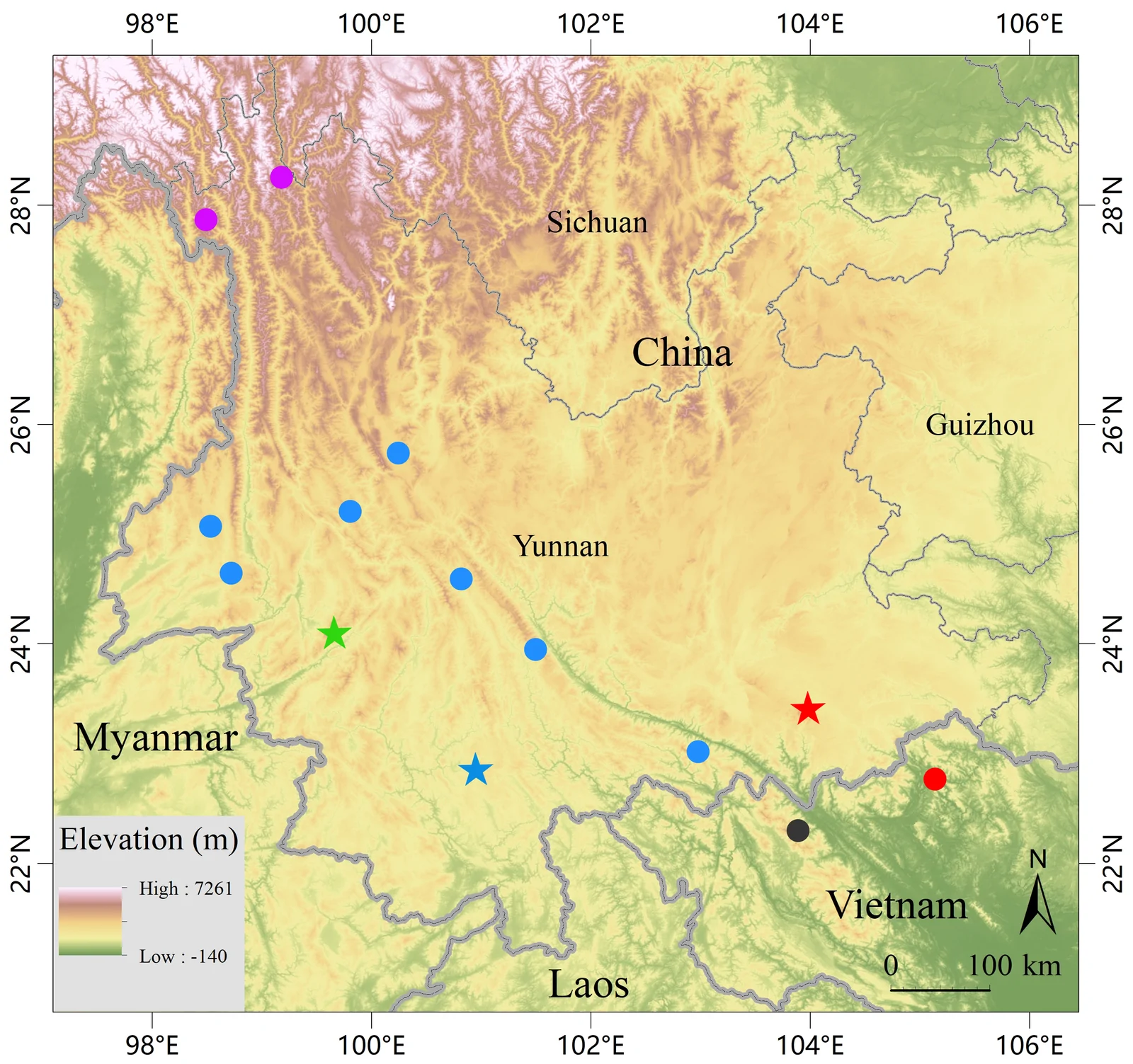
Distributions of *Zhangixalus puerensis* sensu lato. Blue represents Zhangixalus puerensis sensu stricto, purple represents Z. cf. puerensis, red represents *Zhangixalus liui* sp. nov., green represents *Zhangixalus orlovi* sp. nov. and black represents the unnamed species. The stars represent the type localities.

Previously, *Zhangixalus puerensis* was considered to be widely distributed in most parts of Yunnan Province of China and northern Vietnam (Li et al. 2012, AmphibiaChina 2026). In this study, we demonstrated that *Z. puerensis* sensu lato consists of four divergent lineages. We restricted the range of Z*. puerensis* sensu stricto to parts of western, central and southern Yunnan, describing the populations from Yongde County and Wenshan City, Yunnan, China, as two new species (Fig. 11). Two sequences (JN688894 and JN688893) from GenBank were clustered with the sequences of our specimens from Wenshan City, Yunnan, China, with strong support in the phylogenetic analysis. The genetic distances between the two sequences (JN688894 and JN688893) and the sequences of our specimens from Wenshan City, Yunnan, China, was only a negligible 0.5%. Therefore, we regard the specimens corresponding to the sequences (JN688894 and JN688893) from Ha Giang Province of Vietnam and our specimens from Wenshan City, Yunnan, China, as the same species, namely *Zhangixalus liui* sp. nov. Although the population from northwest Vietnam formed a distinct lineage in the phylogenetic analysis, we tentatively refer to this population as an unnamed species as we only obtained the molecular data from GenBank and do not have available specimens of this population. In addition, this study did not obtain any specimen from northern Yunnan; therefore, the taxonomic status of the population recorded as Z*. puerensis* distributed in northwest Yunnan still needs further verification and whether the populations previously considered to be *Z. dugritei* or *Z. hui* distributed in central northern and north-eastern Yunnan involve *Z. puerensis* sensu lato also requires more field surveys and taxonomic studies to clarify.

The genetic distances amongst the four clades of *Zhangixalus puerensis* sensu lato were relatively small; however, they were greater than the genetic distances between some present species in the genus, such as those between *Z. dugritei* and *Z. hui*, between *Z. duboisi* and *Z. meimontis* and between *Z. lishuiensis* and *Z. zhoukaiyae*. Although *Z. hui* may be a synonym of *Z. dugritei*, *Z. duboisi*, *Z. meimontis*, *Z. lishuiensis* and *Z. zhoukaiyae* should be valid species. There is an obvious morphological difference between *Z. duboisi* and *Z. meimontis*, namely one with vocal sacs and the other without, which has traditionally been used as a diagnostics, characteristic in frog species identification (Fei 1999, Fei et al. 2005). Recently, some scholars considered that *Z. lishuiensis* and *Z. zhoukaiyae* are two separate species (e.g. Nguyen et al. (2024), Pan et al. (2024), Pan et al. (2025)). In addition, the genetic distances amongst the four clades of *Z. puerensis* sensu lato were approximately equivalent to those between many present species in the genus. Therefore, the four clades of *Z. puerensis* sensu lato should be treated as four different species. Anyway, the taxonomy of this genus is far from clear. The result of this study is based solely on a mitochondrial gene fragment. In the future, more gene fragments, especially nuclear genes, should be added to obtain more reliable results and further clarify the taxonomic issues of this genus.

Some scholars distinguish different species, based on whether they have internal or external vocal sac (e.g. Mo et al. (2016), Yu et al. (2019), Li et al. (2022), Pan et al. (2024), Pan et al. (2025)). However, there may be some subjectivity in the judgement of the internal or external vocal sac. Although Fei et al. (2009) defines these two types of sacs as those that are directly visible from the outside are external vocal sacs, while those that are not visible from the outside are internal vocal sacs. However, Fei et al. (2009) also mentioned that the wall of the external vocal sac is only composed of skin, while the wall of the internal vocal sac is composed of muscle layer and skin. Therefore, the judgement of the type of vocal sac should not be based only on external observation, but should be carefully examined by checking the walls of the vocal sacs to identify them. Through our observation, we found that it is often difficult to distinguish internal and external vocal sacs solely based on their appearance and there may be some inaccuracies in the previous description of whether the vocal sac of a species is internal or external from literature. Here, we suggest caution in using whether the vocal sac is internal or external to distinguish species.

The collection sites of these two new species are both within nature reserves and their populations are relatively large. Therefore, we consider that they are not currently endangered. Further investigation on the actual distribution range of these two species and the observation of their habits should be strengthened to obtain more ecological information about them.

## Supplementary Material

XML Treatment for Zhangixalus liui

XML Treatment for Zhangixalus orlovi

4958752A-35BD-5226-A076-7FE36D37F46C 10.3897/BDJ.14.e204046.suppl1 22750744 Supplementary material 1 Mean uncorrected pairwise distances Data type table Brief description Mean uncorrected pairwise distances between species of the genus *Zhangixalus* and outgroups based on 16S rRNA sequences. File: oo_1678983.xls https://binary.pensoft.net/file/1678983 Shuo Liu

## Figures and Tables

**Table 1.** Information for voucher numbers, localities and GenBank accession numbers of samples used in molecular phylogenetic analysis.

Species	Voucher No.	Locality	Accession
*Zhangixalus liui* sp. nov.	KIZ2025045	Wenshan, Yunnan, China	PZ945838
*Zhangixalus liui* sp. nov.	KIZ2025046	Wenshan, Yunnan, China	PZ945839
*Zhangixalus liui* sp. nov.	KIZ2025047	Wenshan, Yunnan, China	PZ945840
*Zhangixalus liui* sp. nov.	KIZ2025048	Wenshan, Yunnan, China	PZ945841
*Zhangixalus liui* sp. nov.	KIZ2025049	Wenshan, Yunnan, China	PZ945842
*Zhangixalus liui* sp. nov.	KIZ2025214	Wenshan, Yunnan, China	PZ945843
*Zhangixalus liui* sp. nov.	KIZ2025215	Wenshan, Yunnan, China	PZ945844
*Zhangixalus liui* sp. nov.	KIZ2025216	Wenshan, Yunnan, China	PZ945845
*Zhangixalus liui* sp. nov.	KIZ2025217	Wenshan, Yunnan, China	PZ945846
*Zhangixalus liui* sp. nov.	AMNH 106539	Ha Giang, Vietnam	JN688894
*Zhangixalus liui* sp. nov.	AMNH 106540	Ha Giang, Vietnam	JN688893
*Zhangixalus orlovi* sp. nov.	KIZ2026015	Yongde, Yunnan, China	PZ945847
*Zhangixalus orlovi* sp. nov.	KIZ2026016	Yongde, Yunnan, China	PZ945848
*Zhangixalus orlovi* sp. nov.	KIZ2026017	Yongde, Yunnan, China	PZ945849
*Zhangixalus orlovi* sp. nov.	KIZ2026018	Yongde, Yunnan, China	PZ945850
*Zhangixalus orlovi* sp. nov.	KIZ2026019	Yongde, Yunnan, China	PZ945851
*Zhangixalus orlovi* sp. nov.	KIZ2026020	Yongde, Yunnan, China	PZ945852
*Zhangixalus orlovi* sp. nov.	KIZ2026021	Yongde, Yunnan, China	PZ945853
*Zhangixalus orlovi* sp. nov.	KIZ2026022	Yongde, Yunnan, China	PZ945854
*Zhangixalus orlovi* sp. nov.	KIZ2026023	Yongde, Yunnan, China	PZ945855
*Zhangixalus orlovi* sp. nov.	KIZ2026024	Yongde, Yunnan, China	PZ945856
*Zhangixalus arboreus*	TTU–R–11748	Japan	AF458142
*Zhangixalus burmanus*	SCUM 060614L	Gongshan, Yunnan, China	EU215537
*Zhangixalus chenfui*	SCUM 060404L	Emeishan, Sichuan, China	EU215534
*Zhangixalus daweishanensis*	GXNU YU000392	Pingbian, Yunnan, China	PQ998508
*Zhangixalus dennysi*	SCUM060401L	Shaoguan, Guangdong, China	EU215545
*Zhangixalus dorsoviridis*	ROM 38015	Sa Pa, Lao Cai, Vietnam	JX219423
*Zhangixalus duboisi*	ROM38771	Sa Pa, Lao Cai, Vietnam	JX219414
*Zhangixalus dugritei*	SCUM 051001L	Baoxing, Sichuan, China	EU215541
*Zhangixalus dulitensis*	BORNEENSIS09087	Borneo, Malaysia	AB847123
*Zhangixalus feae*	SCUM 050642W	Hekou, Yunnan, China	EU215544
*Zhangixalus franki*	VNMN 011687	Ha Giang, Vietnam	LC548746
*Zhangixalus hongchibaensis*	CIB 097687	Wuxi, Chongqing, China	JN688883
*Zhangixalus hui*	Li01	Zhaojue, Sichuan, China	JN688878
*Zhangixalus hungfuensis*	SCUM 060425L	Wenchuan, Sichuan, China	EU215538
*Zhangixalus jodiae*	VNMN 07122	Ha Giang, Vietnam	LC545595
*Zhangixalus lishuiensis*	GXNU YU000510	Lishui, Zhejiang, China	PQ901907
*Zhangixalus melanoleucus*	BEI 01010	Phou Samsoum, Xiengkhoang, Laos	OQ305233
*Zhangixalus minimus*	KIZ 061214YP	Jinxiu, Guangxi, China	EU215539
*Zhangixalus moltrechti*	SCUM 061106L	Lianhuachi, Taiwan, China	EU215543
*Zhangixalus nanshanensis*	GXNU YU000771	Chengbu, Hunan, China	PV010198
*Zhangixalus nigropunctatus*	SCUM 070657L	Weining, Guizhou, China	EU215533
*Zhangixalus omeimontis*	SCUM 0606137L	Pengxian, Sichuan, China	EU215535
*Zhangixalus pachyproctus*	KIZ090148	Pu'er, Yunnan, China	MN613222
*Zhangixalus pinglongensis*	NHMG201002011	Shiwandashan, Guangxi, China	KU170684
*Zhangixalus puerensis*	SCUM 060649L	Pu'er, Yunnan, China	EU215542
*Zhangixalus puerensis*	SCUM 060648L	Pu'er, Yunnan, China	JN688884
*Zhangixalus puerensis*	SCUM 0606146L	Tengchong, Yunnan, China	JN688887
*Zhangixalus puerensis*	SCUM 0606147L	Tengchong, Yunnan, China	JN688888
*Zhangixalus puerensis*	SCUM 050421L	Tengchong, Yunnan, China	JN688886
*Zhangixalus puerensis*	SCUM 0606148L	Longling, Yunnan, China	JN688885
*Zhangixalus puerensis*	KIZ060821141	Jingdong, Yunnan, China	EF564542
*Zhangixalus puerensis*	KIZ060821181	Jingdong, Yunnan, China	EF564544
*Zhangixalus puerensis*	KIZ2025135	Changning, Yunnan, China	PZ945857
*Zhangixalus puerensis*	KIZ2024066	Dali, Yunnan, China	PZ945858
*Zhangixalus puerensis*	KIZ2020013	Xinping, Yunnan, China	PZ945859
*Zhangixalus puerensis*	KIZ2023117	Yuanyang, Yunnan, China	PZ945860
*Zhangixalus schlegelii*	–	Hiroshima, Japan	AB202078
*Zhangixalus smaragdinus*	KIZ 20160298	Yingjiang, Yunnan, China	MN613219
*Zhangixalus thaoae*	IEBR A 5136	Lao Cai, Vietnam	LC762092
*Zhangixalus wui*	CIB 097685	Lichuan, Hubei, China	JN688881
*Zhangixalus yaoshanensis*	NHMG150408	Jinxiu, Guangxi, China	MG322122
*Zhangixalus yunnanensis*	GXNU YU20160269	Xinping, Yunnan, China	PV010194
*Zhangixalus zhoukaiyae*	GXNU YU05293	Jinzhai, Anhui, China	PQ901908
*Zhangixalus* sp.	ROM 38640	Sa Pa, Lao Cai, Vietnam	JN688892
*Zhangixalus* sp.	ROM 38641	Sa Pa, Lao Cai, Vietnam	JN688889
*Zhangixalus* sp.	ROM 38647	Sa Pa, Lao Cai, Vietnam	JN688890
*Zhangixalus* sp.	ROM 37996	Sa Pa, Lao Cai, Vietnam	JN688891
*Zhangixalus* sp.	NMN 4100	Sa Pa, Lao Cai, Vietnam	LC010578
*Zhangixalus* sp.	NMN 4101	Sa Pa, Lao Cai, Vietnam	LC010579
*Zhangixalus* sp.	NMN 4102	Sa Pa, Lao Cai, Vietnam	LC010580
*Zhangixalus* sp.	NMN 4103	Sa Pa, Lao Cai, Vietnam	LC010581
*Zhangixalus* sp.	NMN 4104	Sa Pa, Lao Cai, Vietnam	LC010582
*Leptomantis gauni*	FMNH273928	Sarawak, Malaysia	JX219456
*Rhacophorus rhodopus*	SCUM 060692L	Mengyang, Yunnan, China	EU215531
*Theloderma albopunctatum*	ROM 30246	Vietnam	AF458148

**Table 2.** Measurements (in mm) of *Zhangixalus liui* sp. nov. Abbreviations defined in Materials and Methods.

	KIZ2025217 Male Holotype	KIZ2025045 Male Paratype	KIZ2025046 Male Paratype	KIZ2025047 Male Paratype	KIZ2025048 Male Paratype	KIZ2025049 Male Paratype	KIZ2025214 Male Paratype	KIZ2025215 Male Paratype	KIZ2025216 Female Paratype
SVL	45.1	46.4	44.9	44.7	44.6	42.1	43.2	44.5	57.9
HL	16.2	16.0	15.9	15.4	15.5	14.7	14.9	15.4	19.2
HW	17.2	17.5	16.7	16.3	16.9	15.6	16.8	17.1	21.5
SL	7.9	7.5	7.3	7.3	7.6	6.8	6.9	7.3	8.6
IND	5.0	5.0	4.9	4.9	5.0	4.8	4.8	5.1	6.3
IOD	4.7	4.7	4.4	4.3	4.8	4.1	4.2	4.6	5.4
UEW	4.3	3.9	3.8	4.1	3.6	3.6	3.9	4.1	4.5
ED	4.9	5.0	4.9	4.9	4.8	4.7	4.8	4.6	5.3
TD	2.9	3.1	2.7	2.7	2.9	2.6	2.7	2.8	4.6
FHL	23.9	24.0	23.8	24.0	23.7	23.2	24.3	24.3	32.7
TL	18.5	18.7	17.9	18.0	18.5	17.2	18.0	17.7	25.3
TFL	29.9	29.8	29.3	29.6	29.2	27.9	29.6	28.3	40.6
FL	20.5	20.7	20.9	20.5	19.6	18.8	21.1	20.0	27.5

**Table 3.** Measurements (in mm) of *Zhangixalus orlovi* sp. nov. Abbreviations defined in Materials and Methods.

	KIZ2026024 Male Holotype	KIZ2026015 Male Paratype	KIZ2026016 Male Paratype	KIZ2026017 Male Paratype	KIZ2026018 Male Paratype	KIZ2026019 Male Paratype	KIZ2026020 Female Paratype	KIZ2026021 Female Paratype	KIZ2026022 Male Paratype	KIZ2026023 Male Paratype
SVL	41.3	40.9	38.3	34.6	38.2	34.0	51.2	54.3	38.7	40.6
HL	14.1	14.4	14.0	12.4	13.5	11.9	17.2	18.2	13.4	13.8
HW	14.7	14.9	14.5	12.8	13.9	13.2	19.1	19.8	14.2	14.7
SL	6.6	6.7	6.8	6.1	6.4	5.6	7.5	8.1	6.2	6.7
IND	4.7	4.6	4.6	4.0	4.6	3.9	5.6	5.9	4.3	4.6
IOD	4.2	4.0	4.0	3.7	4.1	3.4	5.1	5.3	4.1	4.0
UEW	3.4	3.7	3.5	3.4	3.2	3.0	4.2	4.4	3.2	3.6
ED	4.5	4.6	4.1	3.6	4.1	3.9	4.9	5.3	4.2	4.6
TD	2.4	2.2	2.3	1.9	2.3	1.8	3.0	3.9	2.2	2.3
FHL	21.4	21.7	20.1	19.1	19.7	18.4	28.6	29.0	20.5	20.9
TL	16.4	16.9	16.0	15.1	16.6	14.9	22.4	24.6	15.4	16.1
TFL	26.7	26.3	25.0	23.2	24.4	23.3	35.6	39.1	25.0	26.5
FL	18.8	17.5	17.4	15.4	16.3	15.8	24.7	26.9	17.2	18.6
